# Triboelectric Plasma CO_2_ Reduction Reaching a Mechanical Energy Conversion Efficiency of 2.3%

**DOI:** 10.1002/advs.202201633

**Published:** 2022-06-09

**Authors:** Sumin Li, Bao Zhang, Guangqin Gu, Dongyang Fang, Xiaochen Xiang, Wenhe Zhang, Yifei Zhu, Jiao Wang, Junmeng Cuo, Peng Cui, Gang Cheng, Zuliang Du

**Affiliations:** ^1^ Key Lab for Special Functional Materials, Ministry of Education National & Local Joint Engineering Research Center for High‐efficiency Display and Lighting Technology School of Materials Science and Engineering and Collaborative Innovation Center of Nano Functional Materials and Applications Henan University Kaifeng 475004 China; ^2^ Institute of Aero‐engine School of Mechanical Engineering Xi'an Jiaotong University Xi'an 710049 P. R. China

**Keywords:** chemical energy, CO_2_ reduction, energy conversion efficiency, mechanical energy, triboelectric plasma

## Abstract

Mechanical energy‐induced CO_2_ reduction is a promising strategy for reducing greenhouse gas emissions and simultaneously harvesting mechanical energy. Unfortunately, the low energy conversion efficiency is still an open challenge. Here, multiple‐pulse, flow‐type triboelectric plasma with dual functions of harvesting mechanical energy and driving chemical reactions is introduced to efficiently reduce CO_2_. CO selectivity of 92.4% is achieved under normal temperature and pressure, and the CO and O_2_ evolution rates reach 12.4 and 6.7 µmol h^−1^, respectively. The maximum energy conversion efficiencies of 2.3% from mechanical to chemical energy and 31.9% from electrical to chemical energy are reached. The low average electron energy in triboelectric plasma and vibrational excitation dissociation of CO_2_ with low barrier is revealed by optical emission spectra and plasma simulations, which enable the high energy conversion efficiency. The approach of triboelectric plasma reduction reported here provides a promising strategy for efficient utilization of renewable and dispersed mechanical energy.

## Graphical abstract

Triboelectric plasma creates simultaneously harvesting mechanical energy and driving CO_2_ reduction to CO, efficiently increasing the overall energy efficiency from mechanical to chemical energy. Moreover, this system shows well‐suited to the irregularity and volatility of mechanical energy and the flow of CO_2_ gas. This strategy exhibits potential in mechanical energy storage and mechanical energy‐driven CO_2_ reduction.

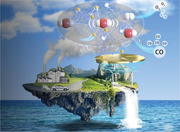

## 1 Introduction

Low‐frequency mechanical energy is abundant, clean, and renewable and can be harvested from sources such as wind energy, water energy, and ocean wave energy.^[^ ^1^ ^]^ Unfortunately, the storage of irregular, intermittent, fluctuating mechanical energy is challenging, which hinders its effective transformation and utilisation.^[^ ^2^ ^]^ An ideal method to store mechanical energy is to use it to produce chemical fuels^[^ ^3^ ^]^ as they are considered green mechanical energy carriers. In this context, the most appealing method is the use of mechanical energy to reduce CO_2_ emissions because it can solve the problem of mechanical energy storage and reduce global carbon emissions, alleviating the environmental damage caused by greenhouse gases.^[^ ^4^ ^]^ However, the mechanical energy‐induced reduction of chemically inert CO_2_ molecules is difficult at room temperature and pressure.^[^ ^5^ ^]^ Moreover, reports on the direct use of mechanical energy to convert CO_2_ are insufficient.

The conversion of low‐frequency mechanical energy into electrical energy using a triboelectric nanogenerator (TENG) has gained popularity in recent years.^[^ ^6^ ^]^ The electrochemical CO_2_ reduction systems have also been proposed by combining TENGs and electrochemical reactions, which directly collect mechanical energy and drive electrochemical reactions. However, due to the mismatch of the low required voltage of electrochemical reaction (≈V) and the high output voltage of TENG (≈kV), the power management and storage units are introduced in these systems, resulting in a lot of energy losses and low energy conversion efficiency from mechanical to chemical energy (0.5‰ in an electrochemical CO_2_ reduction system powered by ocean wave energy).^[^ ^7^ ^]^ It has been reported that the high output voltage of TENGs enables the generation of triboelectric plasma from a gas discharge at normal temperature and pressure.^[^ ^8^ ^]^ Previously, Li et al. constructed a static, mono‐pulse corona type triboelectric plasma for CO_2_ reduction, and achieved a conversion efficiency of 5.2% from electrical to chemical energy.^[^ ^8a^ ^]^ However, the energy conversion efficiency is still low, which is attributed to the high average energy of electrons in the triboelectric plasma and CO_2_ decomposition via a high‐energy‐barrier pathway. Therefore, to achieve a higher energy conversion efficiency, it is urgent to conduct triboelectric plasma CO_2_ reduction through a low‐energy‐barrier pathway.^[^ ^9^ ^]^ Moreover, as a type of nonthermal micro‐plasma, triboelectric plasma could simultaneously harvest mechanical energy and drive CO_2_ reduction reactions, which could be utilized to develop mechanical energy‐induced CO_2_ reduction system. This system has the advantages of high efficiency and low cost, since the triboelectric plasma is produced by renewable and dispersed mechanical energy, and no power management and energy storage units are needed in the system.^[^ ^10^ ^]^

Herein, a mechanical energy‐induced CO_2_ reduction system was proposed based on a dual‐function, multiple‐pulse, flow‐type triboelectric plasma, which simultaneously harvested mechanical energy and drove CO_2_ reduction reactions. The system was well‐suited to the instabilities of mechanical energy and the fluctuating CO_2_ gas flow. As the discharge distance was 0.8 mm, CO and O_2_ evolution rates of 12.4 and 6.7 µmol h^−1^, respectively, were achieved. The CO selectivity was 92.4%. The conversion efficiency from electrical to chemical energy (*η*_ele − chem_) was 31.9%; this value is higher than those previously reported for nonthermal plasma‐triggered CO_2_ reduction systems. The maximum energy conversion efficiency from mechanical to chemical energy (*η*_mech − chem_) was 2.3%, the highest value reported to date. We investigated the pathway of CO_2_ dissociation using plasma simulation and optical emission spectroscopy. Finally, field experiments were conducted at a wind speed of 4.0 m s^−1^, achieving the maximum CO yield of 16.8 µmol h^−1^. Our work demonstrates a potential candidate for efficiently converting mechanical energy into chemical energy.

## 2 Results and Discussion

### 2.1 CO_2_ Reduction Using Dual‐Function, Multiple Pulse, Flow‐Type Triboelectric Plasma Generated by Mechanical Energy

**Figure 1:**
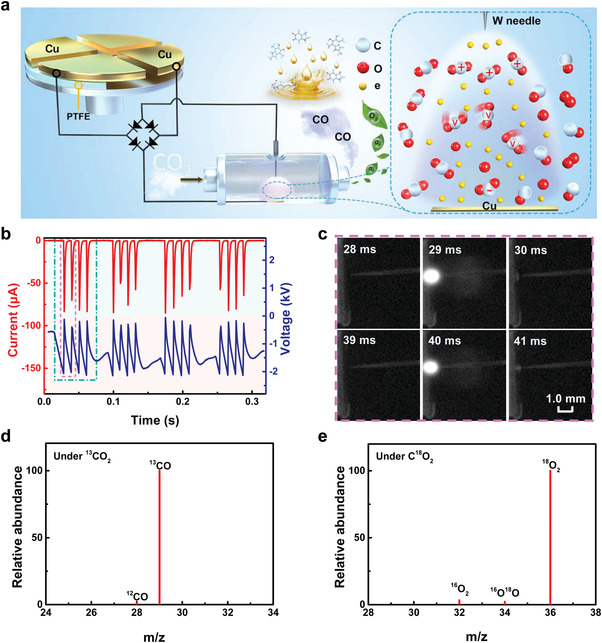
Mechanical energy‐induced CO_2_ reduction system driven by dual‐function, multiple‐pulse, flow‐type triboelectric plasma. a) Schematic of the experimental device. b) Current and voltage curves of the triboelectric plasma versus time. c) High‐speed photographs of the triboelectric plasma. d) ^13^CO_2_‐labeled mass spectrum of CO product. e) C^18^O_2_‐labeled mass spectrum of O_2_ product. Reaction conditions: discharge distance, 0.8 mm; TENG rotational speed, 180 rpm; CO_2_ flow rate, 10.0 mL min^−1^; room temperature; and atmospheric pressure.

Our CO_2_ reduction system based on dual‐function, multiple‐pulse, flow‐type triboelectric plasma is shown in **Figure** **1**a. The system comprised two parts: a TENG and a triboelectric plasma‐induced CO_2_ reduction reactor. The TENG was used to collect mechanical energy from nature and convert it into electrical energy to generate the triboelectric plasma. The plasma CO_2_ reduction reactor comprised a flow‐type needle‐plate gas‐discharge device for CO_2_ reduction. When polytetrafluoroetheylene (PTFE) and Cu films came in contact, negative and positive triboelectric charges were generated on the PTFE and Cu surfaces, respectively, due to the difference in triboelectric sequence. There was a periodic potential difference between the two groups of Cu electrodes of the TENG when the PTFE was rotated and the open‐circuit voltage reached 4.8 kV (Figure S1, Supporting Information). When the output voltage of the TENG exceeded the threshold voltage of CO_2_ gas, the gas contact between the needle and the plate was ionized, producing the triboelectric plasma. Figure 1b illustrates the electrical curve of triboelectric plasma when the discharge distance (*d*) was 0.8 mm and the TENG rotational speed was 180 rpm. In half a cycle, four discharge voltage peaks (*V*) of ≈2.1 kV each and four discharge current peaks (*I*) of ≈79.1 µA each were generated (green dashed frame in Figure 1b); this corresponds to the generation of triboelectric plasma. The type of triboelectric plasma is a multiple‐pulse, flow‐type discharge, which is different from the static, mono‐pulse corona discharge previously reported in the literature.^[^ ^8a^ ^]^ The discharge process is shown in Figure 1c and Figure S2, Supporting Information. The triboelectric plasma formation process was divided into three stages: before, during, and after the breakdown (Figure 1c). A single pulse lasted ≈1.0 ms, and the length of the triboelectric plasma was 0.8 mm. According to ^13^C and ^18^O‐labeled isotope experiments, the triboelectric plasma generated by mechanical energy‐reduced CO_2_ to produce CO and O_2_ (Figure 1d,e).

### 2.2 Influence of Mechanical Energy Output and CO_2_ Flow Rate on CO_2_ Reduction

**Figure 2:**
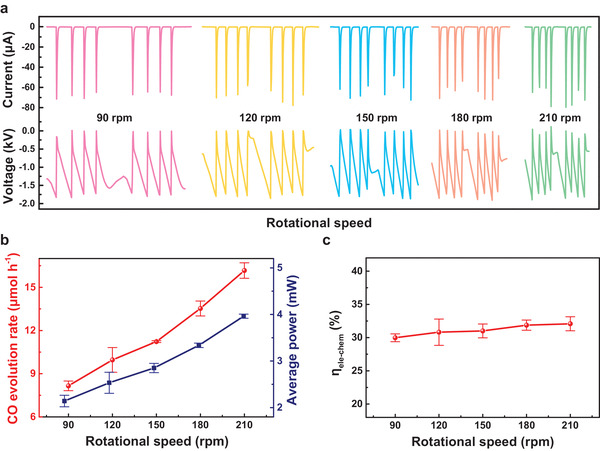
Influence of the TENG rotational speed on the CO_2_ decomposition performance. a) Current and voltage curves at different TENG rotational speeds. b) CO evolution rate and average power versus TENG rotational speed. c) Conversion efficiency of electrical to chemical energy (*η*_ele − chem_) versus TENG rotational speed. Reaction conditions: discharge distance, 0.8 mm; CO_2_ flow rate, 10.0 mL min^−1^; room temperature and atmospheric pressure.

We investigated the influence of the TENG rotational speed on the CO_2_ reduction performance. The frequency of triboelectric plasma increased with the rotational speed (**Figure** **2**a), but the number of discharge pulses, *V*, and *I* remained constant during half a cycle (Figure S3, Supporting Information). These results imply that approximately the same state of triboelectric plasma was formed at different rotational speeds. The average power (*P*_ave_) of the triboelectric plasma increased from 2.1 to 4.0 mW and the CO evolution rate (*r*_CO_) increased from 8.2 to 16.2 µmol h^−1^ as the rotational speed increased from 90 to 210 rpm (Figure 2b). *η*_ele − chem_ was 30.0–31.9% (Figure 2c). These results suggest the good environmental compatibility and adaptability of the present mechanical energy‐driven CO_2_ reduction system.

We also investigated the effect of the CO_2_ flow rate on the reduction performance. The frequency of triboelectric plasma, *V*, *I*, and *P*_ave_ values remained constant upon increasing the CO_2_ flow rate from 0.2 to 12.5 mL min^−1^, indicating that approximately the same state of triboelectric plasma was formed at different CO_2_ flow rates (Figures S4 and S5, Supporting Information). Moreover, when the CO_2_ flow rate increased from 0.2 to 12.5 mL min^–1^, the *r*_CO_ and *η*_ele − chem_ were all the same, which is indicative of the excellent flow rate compatibility of the system. Furthermore, the conversion rate of CO_2_ decreased from 2.74% to 0.04% as the CO_2_ flow rate increased from 0.2 to 12.5 mL min^–1^. At a CO_2_ flow rate of 10.0 mL min^–1^, the conversion rate of 0.05% was achieved.

### 2.3 Influence of *d* on CO_2_ Reduction

**Figure 3:**
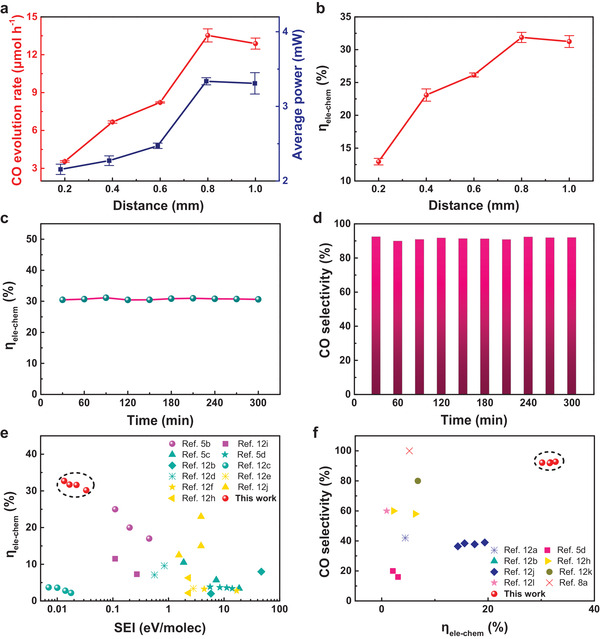
Influence of the discharge distance on the CO_2_ decomposition performance. a) CO evolution rate and average power versus discharge distance. b) Conversion efficiency of electrical to chemical energy (*η*_ele − chem_) versus discharge distance. c) *η*_ele − chem_ within 300 min of reaction at a discharge distance of 0.8 mm. d) CO selectivity within 300 min of reaction at a discharge distance of 0.8 mm. Reaction conditions: TENG rotational speed, 180 rpm; flow rate, 10.0 mL min^−1^; room temperature and atmospheric pressure. e) *η*_ele − chem_ as a function of the molar ratio of specific energy input (SEI) to reaction gas in different types of nonthermal plasma measured for CO_2_ conversion. f) CO selectivity versus *η*_ele − chem_ in different types of nonthermal plasma measured for CO_2_ conversion.

Next, we investigated the effect of the *d* value. As *d* increased from 0.2 to 1.0 mm, the number of discharge pulses decreased from 7 to 3, *V* increased from 1.5 to 2.3 kV, and *I* increased from 60.2 to 81.8 µA (Figure S6, Supporting Information). The increase in *d* hinders the formation of a conductive pathway between the needle and the plate electrode. Therefore, a higher threshold voltage was required to achieve the gas discharge and the number of discharges in each cycle decreased. Consequently, the amount of charge per discharge pulse increased, resulting in a higher discharge current. As illustrated in **Figure** **3**a, as *d* increased, *P*_ave_ increased from 2.1 to 3.3 mW and *r_CO_* increased from 3.5 to 12.9 µmol h^−1^. Since the increase in *r_CO_* was more pronounced than that in *P*_ave_, the *η*_ele − chem_ value increased with increasing *d* (Figure 3b), reaching an optimal value of 31.9% for a *d* of 0.8 mm. Further increasing *d* to 1.0 mm decreased the *η*_ele − chem_ value to 31.2%. The discharge changed from multiple pulse discharges to mono pulse, corona discharges as *d* increased to 1.2 mm (Figure S6, Supporting Information).^[^ ^8a^ ^]^ The results show that the pathway of CO_2_ reduction driven by mechanical energy‐generated triboelectric plasma varies significantly with *d*. To test the stability of the triboelectric plasma reduction system, we conducted the CO_2_ reduction reaction for 5 h continuously at a rotational speed of 180 rpm, a *d* of 0.8 mm, and a gas flow rate of 10.0 mL min^−1^, affording an *r_CO_* of 12.1–12.3 µmol h^−1^, an O_2_ generation rate of 6.6–6.7 µmol h^−1^, an *η*_ele − chem_ of 30.5–31.9%, and a CO selectivity of 90.8–92.4% (Figure S7, Supporting Information and Figure 3c,d). These results demonstrate the good long‐term stability of the system.

Triboelectric plasma is a type of nonthermal plasma that is driven by mechanical energy.^[^ ^11^ ^]^ To demonstrate the benefits of using triboelectric plasma for CO_2_ reduction over other types of nonthermal plasma, such as dielectric barrier discharge and nanosecond pulse corona, we compared the energy conversion efficiencies and selectivities of the present CO_2_ reduction system with those previously reported.^[^ ^5^, ^12^ ^]^ As shown in Figure 3e,f, our system afforded an *η*_ele − chem_ of 31.9% for a molar ratio of specific energy input (SEI) to reaction gas of 0.1 eV molec^−1^, outperforming other nonthermal plasmas (Table S1, Supporting Information). The selectivity for CO was 92.4%, which is higher than most of these results reported in the literature (Table S2, Supporting Information).

Next, we measured the power of mechanical energy provided for the TENG (*P*_mech_) via dynamic torque measurement and the CO yield. *η*_mech − chem_ was ≈2.1–2.3% (Figure S8, Supporting Information). The maximum *η*_mech − chem_ value was 46 times higher than that reported for a mechanical energy‐driven CO_2_ reduction system tested in a natural environment.^[^ ^7^ ^]^ Specifically, the *η*_mech − chem_ of an electrochemical reduction CO_2_ system driven by ocean wave energy was only 0.5‰, as shown in Table S3, Supporting Information. The low energy conversion efficiency of the ocean wave energy‐driven electrochemical reduction CO_2_ system is due to a mismatch between the low required voltage (≈V) of the electrochemical reaction device and the high output voltage (≈kV) of TENG. In our system, the triboelectric plasma could effectively decrease the impedance of TENG, enhancing the TENG output and improving the efficiency of the entire mechanical energy‐driven CO_2_ reduction system.^[^ ^13^ ^]^

### 2.4 Mechanism of CO_2_ Reduction Driven by Mechanical Energy‐Generated Triboelectric Plasma

**Figure 4:**
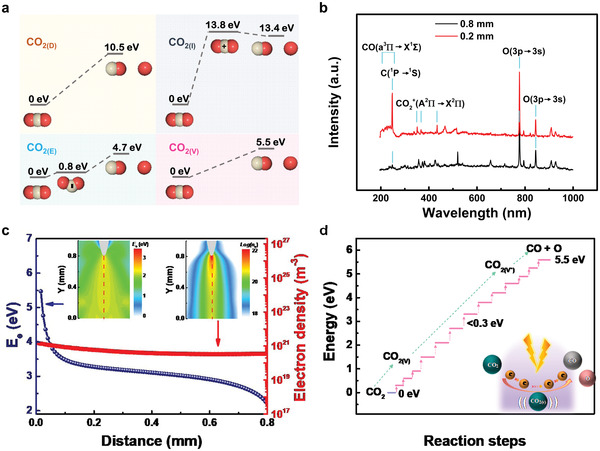
Mechanism of triboelectric plasma‐driven CO_2_ reduction. a) Diagrams of four plausible dissociation pathways of CO_2_ decomposition in the plasma. b) Optical emission spectra of the triboelectric plasma with discharge distances of 0.2 and 0.8 mm. c) Average electron energy (*E*_e_) and electron density (*n*_e_) of mid perpendicular at an evolution time of 6.0 ns and a discharge distance of 0.8 mm. d) Schematic illustration of the vibrational excitation dissociation pathway. Note that CO_2(v)_ and CO_2(v*)_ stand for different vibrationally excited levels, being CO_2(v*)_ at a higher level than CO_2(v)_.

We carefully analyzed the CO_2_ decomposition pathway to understand the reason for the high energy conversion efficiency and selectivity of CO_2_ reduction via triboelectric plasma. According to the literature, the conversion of CO_2_ into CO can proceed via four mechanisms, that is, electronic excitation dissociation (CO_2(D)_), electron impact ionization (CO_2(I)_), electron attachment dissociation (CO_2(E)_), and vibrational excitation dissociation (CO_2(V)_),^[^ ^14^ ^]^ whose intermediates and energy barriers differ significantly (**Figure** **4**a and Table S4, Supporting Information). Thus, the CO_2(D)_ and CO_2(I)_ pathways exhibit high energy barriers of 10.5 and 13.8 eV, respectively, whereas the CO_2(E)_ and CO_2(V)_ pathways exhibit low energy barriers of 4.7 and 5.5 eV, respectively.

The optical emission spectra (OES) of the intermediates or products in the four dissociation processes can help elucidate the mechanism of CO_2_ dissociation.^[^ ^15^ ^]^ The emission spectra measured using a QEpro‐High Performance Spectrometer at *d* values of 0.2 and 0.8 mm are shown in Figure 4b. At a *d* of 0.2 mm, a medium‐intensity spectral line attributable to the transition of C atoms from the ^1^P state to the ^1^S state was produced at 247 nm, indicating the occurrence of a high‐energy consumption process of CO_2_ decomposition into C. This line almost vanished at a *d* of 0.8 mm, suggesting that CO_2_ decomposition into C ceased. A set of broad emission bands was observed at 206–258 nm at a *d* of 0.2 mm, which can be attributed to the excited‐state transition of CO from the a^3^П state to the X^1^Σ state (Table S5, Supporting Information). These bands are in agreement with the CO_2(D)_ pathway since this is the only mechanism that proceeds via excited‐state CO, whereas the other three pathways involve ground‐state CO. These bands almost vanished at a *d* of 0.8 mm, indicating the disappearance of the energy‐intensive CO_2(D)_ pathway. The formation of triboelectric plasma requires the generation of CO_2_^+^ ions, whose dissociation involves no potential barrier. Therefore, the CO_2(I)_ pathway cannot be avoided. At *d* of 0.2 and 0.8 mm, three bands were observed at 351.1, 367.4, and 434.2 nm, which correspond to the transition from the A^2^П state to the X^2^П state of the intermediate CO_2_^+^ ions in the CO_2(I)_ process. Two strong O atoms transitioned from the 3p state to the 3s state, producing bands at 777.5 and 844.7 nm, respectively. This spectrum represents the excited O atoms produced by the CO_2(I)_ and CO_2(V)_ processes. The intermediate CO_2_^−^ ions in the CO_2(E)_ pathway have a very short lifetime and decompose before producing luminescence, which renders them undetectable by conventional OES.^[^ ^16^ ^]^ Using 5,5‐dimethyl‐1‐pyrroline‐*N*‐oxide (DMPO) as a capture reagent, the intermediate CO_2_^−^ species was detected by electron paramagnetic resonance (EPR) spectroscopy (Figure S9, Supporting Information).^[^ ^17^ ^]^ Notably, the dissociation of CO_2_^−^ ions needs to overcome a potential barrier of 3.9 eV. Therefore, the presence of CO_2_^−^ ions indicates the possibility of the CO_2(E)_ pathway. The CO_2(E)_ pathway is determined by two factors: the generation of CO_2_^−^ ions and the energy provided by the triboelectric plasma. The above results suggest that compared with the *d* of 0.2 mm, the two high‐energy dissociation pathways (CO_2(D)_ and the direct decomposition of CO_2_ into C) almost disappear at the *d* of 0.8 mm. Hence, CO_2_ dissociation could proceed via the CO_2(I)_, CO_2(E)_, and CO_2(V)_ pathways.

Considering that the energy and spatial distribution of electrons in the plasma are critical factors in determining the CO_2_ decomposition pathway,^[^ ^18^ ^]^ we calculated the electron density (*n_e_*) and average electron energy (*E_e_*) at various spatial positions during the evolution of a simulated triboelectric plasma with time at different *d* values. The results are shown in Figure S10, Supporting Information. A cylindrical plasma channel formed in the needle‐plate gap as *d* increased from 0.2 to 1.0 mm. The plasma channel had the greatest volume at the *d* of 0.8 mm (the height of the cylindrical area was 0.8 mm and the diameter was 0.3 mm). With increasing *d*, the *n_e_* in the plasma channel decreased, the channel volume increased, and the total number of electrons remained virtually unaltered (Figure S11, Supporting Information). As *d* increased, *E*_e_ gradually decreased (Figure S10c, Supporting Information). When *d* increased from 0.2 to 0.8 mm, *E_e_* of all electrons in the plasma channel decreased from 12.0 to 2.5 eV, which disfavors the high energy‐consuming reaction pathways. This result is consistent with the OES results.

When *d* was 0.8 mm, the triboelectric plasma was generated within 6.0 ns (Figure S12, Supporting Information). In the following 1 ms, the electrons in the triboelectric plasma migrated to the plate electrode to complete the discharge process. Strong electric fields and high‐energy electrons were present in the space during the formation of the triboelectric plasma in the first 6.0 ns. The maximum reduced electric field strength (E/N) was 800 Td and the maximum *E_e_* was 10.5 eV. In this process, a large amount of CO_2_^+^ ions and electrons were produced by avalanche ionization. At an evolution time of 6.0 ns, when the plasma was formed, the E/N in the plasma channel decreased below 100 Td and *E_e_* was also significantly reduced. As shown in Figure 4c, the curve of *E_e_* and *n_e_* on the central axis of the channel varies with the distance. The electron density changed slightly from 1.5 × 10^21^ to 3.5 × 10^20^ m^−3^. From the tip to the plate electrode, *E_e_* decreased from 5.5 to 2.2 eV. Only 0.3% of the electrons had energy exceeding 13.8 eV at a distance of 12.0 µm from the tip, according to the electron energy distribution function (EEDF) (Figure S13, Supporting Information). Therefore, after the plasma channel was formed, almost no new CO_2_^+^ ions were produced. This indicates that CO_2_^+^ ions, the intermediates of the CO_2(I)_ pathway, are mainly produced during the plasma formation process (Figure S14, Supporting Information). When the distance from the tip was 50.0 µm, *E_e_* decreased to 3.9 eV (the energy barrier of the CO_2(E)_ pathway), as shown in Figure 4c. At a distance of 0.1 mm from the tip, ≈5.5% of the electrons had energy exceeding 3.9 eV (Figure S15, Supporting Information). These findings indicate that after the plasma formation, the CO_2(E)_ pathway occurs primarily within 0.1 mm of the needle tip. The CO_2(V)_ pathway gradually excites the ground‐state CO_2_ molecules until it overcomes the energy barrier of CO_2_ decomposition (Figure 4d and Table S6, Supporting Information). Although the total potential barrier reaches 5.5 eV, which is higher than that of the CO_2(E)_ process, the energy required for each excitation step is less than 0.3 eV. Consequently, in the CO_2(V)_ pathway, CO_2_ molecules could use all the low energy electrons in the plasma channel to dissociate into CO and O in the most energy‐efficient manner. According to the above analysis, the CO_2(I)_ process occurs only during the plasma channel formation, whereas the CO_2(E)_ and CO_2(V)_ processes can still occur within the following 1 ms. In terms of spatial distribution, the CO_2(E)_ process mainly occurs in the area near the tip, whereas the CO_2(V)_ process can occur in the entire plasma channel.

The triboelectric plasma driven by mechanical energy has a lower *E_e_* (less than 3.0 eV) than traditional nonthermal plasma (for example, the *E_e_* of DBD is between 3.0 and 15.0 eV),^[^ ^16c^ ^]^ which is suitable for the CO_2(V)_ pathway with low energy and disfavors the high energy‐consuming CO_2(D)_ pathway. Consequently, the *η*_ele − chem_ for CO_2_ reduction is extremely high. Simultaneously, the low *E_e_* hinders the high energy‐consuming direct dissociation of CO_2_ into C and improves the selectivity of the reaction.

### 2.5 Field Experiment and Performance Comparison of CO_2_ Reduction Driven by Triboelectric Plasma

The present CO_2_ reduction system can convert CO_2_ to CO with high efficiency and selectivity and efficiently collect mechanical energy from nature and convert it into chemical energy.^[^ ^19^ ^]^ We measured the amount of CO product through our system in the environment at a wind speed of 4.0 ± 0.2 m s^−1^, finding that after four consecutive cycles, *r_CO_* was 12.8–16.8 µmol h^−1^ (Figures S16,S17, Supporting Information).

Our CO_2_ reduction system is superior to common thermal catalytic and photocatalytic CO_2_ reduction methods because it achieves high energy conversion efficiency in a sustainable manner. Although thermal catalytic processes achieve high energy conversion efficiency (up to 47.0%), they highly depend on fossil fuels.^[^ ^20^ ^]^ Meanwhile, the photocatalytic CO_2_ reduction utilizes renewable, green solar energy, albeit with an energy conversion efficiency below 1.0%.^[^ ^21^ ^]^ Conversely, the present system uses abundant mechanical energy from nature, is easy to operate, does not require catalysts or additional reagents, and can be turned on and off on demand, rendering it suitable for decentralized carbon fixation.

## 3 Conclusion

This work demonstrated a mechanical energy‐induced CO_2_ reduction system using dual‐function, multiple‐pulse, flow‐type triboelectric plasma that operates under mild conditions to realize the collection and conversion of mechanical energy to chemical energy. This system was well‐suited to the fluctuation of mechanical energy and the flow of CO_2_ gas. The CO and O_2_ evolution rates were 12.4 and 6.7 µmol h^–1^, respectively, at an optimal discharge distance of 0.8 mm. The CO selectivity was 92.4%. *η*_ele − chem_ was 31.9%; this value is higher than those reported for CO_2_ reduction methods using nonconvertible energy and other nonthermal plasma systems. The maximum *η*_mech − chem_ was 2.3%, the highest value reported to date. Plasma simulations and OES tests revealed that the average energy of electrons in the triboelectric plasma was low and CO_2_ reduction was primarily accomplished via vibrational excitation dissociation with a low energy barrier. Finally, our system was tested in the environment using wind as a mechanical energy source, obtaining a maximum *r_CO_* of 16.8 µmol h^−1^.

## 4 Experimental Section

### Fabrication of the TENG

The free‐rotating TENG comprised two parts: an electrode stator and a rotator (Figure 1a). A copper foil (60.0 µm thick) was electroplated to a printed circuit board (0.3 cm thick, Ф = 25.0 cm) to construct the stator. The copper foil was evenly divided into four sectors, which were connected by the inner ring or outer ring of the two electrodes in the stator at intervals. The distance between two adjacent sectors of the copper film was adjusted to 1.0 cm. The diameter of the rotator (0.4 cm thick) was 25.0 cm. As previously stated, the two sectors were stacked on the side of a rotator and were composed of PTFE film (0.8 mm thick) and polymethyl methacrylate film. The rotator was connected to the motor shaft via a flange‐mount shaft collar.

### Mechanical Energy‐Induced CO_2_ Reduction Driven by Multiple Pulse, Flow‐Type Triboelectric Plasma

The multiple‐pulse, flowing triboelectric plasma reactor is shown in Figure 1a. A copper foil (0.5 cm × 0.5 cm) was attached to the inside of a glass reactor (external diameter, 1.0 cm; internal diameter, 0.8 cm) as an electrode. A tungsten needle, with a curvature radius of 5 µm, served as the other electrode, which was placed on opposite sides of the glass reactor. Flexible tubing (internal diameter, 0.3 cm) was used to feed CO_2_ gas into one end of the glass reactor and the other end was used for the gas inlet extending to a gas chromatograph. High‐speed photographs of the triboelectric plasma were obtained using high‐speed cameras (TMX7510, Phantom, York Technologies Ltd.).

The gaseous products (CO and O_2_) were detected by an online gas chromatograph (Agilent 7890B) equipped with a Shincarbon column (column temperature, 90 °C), a thermal conductivity detector, and a flame ionization detector with a mechanized oven. Exceptionally pure He served as the transport gas. The concentration of gaseous products was calibrated using standard curves from standard gases. The conversion rate of CO_2_ was estimated as follows.

(1) $$\mathrm{Conversion}\mathrm{rate}\left(\%\right)=\frac{n_{\mathrm{CO}}}{n_{CO_{2}}}$$

where *n*_CO_ is the mole of CO product per time, and $n_{CO_{2}}$ is the initial mole of CO_2_ substrate per time.

### Estimation of *η*_ele − chem_

The discharge voltage and current were measured using two programmable electrometers (Keithley 6514) at different ranges. Before connecting it to the circuit, the electrometer measuring the discharge voltage was connected in series with the appropriate sampling resistor (≈GΩ). The measured signals were fed into a high‐speed data acquisition system controlled by LabView software.

*P*_ave_ was calculated using the following equation.

(2) $$P_{\mathrm{ave}}=\frac{\int_{0}^{t}V\cdot I\cdot dt}{t}$$

where *V* is the discharge voltage (V), *I* is the discharge current (A), and *t* is the discharge time (s).

*η*_ele − chem_ was estimated as follows.

(3) $$\eta_{\mathrm{ele}-\mathrm{chem}}=\frac{r_{\mathrm{CO}}\cdot \Delta H_{R}}{P_{\mathrm{ave}}\cdot 3600}$$

where Δ*H*_R_ is the reaction enthalpy of CO_2_ splitting to CO (279.8 kJ mol^−1^).

### Estimation of *η*_mech − chem_

To accurately measure the *P*_mech_ provided for the TENG, a dynamic torque measurement system was constructed. The fabrication of the TENG used to measure *η*_mech − chem_ was different from that of the TENG used to determine the activity of CO_2_ dissociation at different rotational speeds and needle‐plate distances. The former TENG was constructed using triboelectric layers of rabbit hair and PTFE because the two triboelectric layers were easy to connect to the dynamic torque measurement system. In fact, no significant differences were observed in the energy conversion efficiencies of the CO_2_ reduction systems using the two TENGs. The *P*_mech_ provided for the TENG was accurately measured using the torque sensor (Figure S8, Supporting Information). Simultaneously, the CO yield was determined using the online gas chromatograph.

The *P*_mech_ provided for the TENG was calculated as follows.

(4) $$P_{\mathrm{mech}}=\frac{T\cdot n}{9549}$$

where *T* is the torque (N m) and *n* is the rotational speed of the TENG (r min^−1^).

The following equations were used to calculate *η*_mech − chem_.

(5) $$\eta_{\mathrm{mech}-\mathrm{chem}}=\frac{r_{\mathrm{CO}}\cdot \Delta H_{R}}{P_{\mathrm{mech}}\cdot 3600}$$

where the Δ*H*_R_ value is the same as that described in Equation (3).

### Field Test

To confirm whether the triboelectric plasma‐triggered CO_2_ reduction system can effectively utilize the mechanical energy from nature, field tests at a wind speed of 3.8–4.2 m s^−1^ were performed using a TENG powered by a cup‐shaped wind sensor. The CO_2_ reduction driven by natural wind was conducted in a sealed glass reactor (internal diameter, 8.0 cm; height, 13 cm) and the CO sensor was used for online detection. The maximum range of the CO sensor was 1000 ppm; therefore, the experiment was terminated when the CO concentration in the reactor reached 1000 ppm. The experiment was repeated four times.

### Isotope Labeling and EPR Experiments

^13^C‐ and ^18^O‐isotope labeling experiments were performed using ^13^CO_2_ or C^18^O_2_ as substrates under the same conditions used in previous triboelectric plasma‐triggered CO_2_ reduction reactions. The isotopic products produced by the triboelectric plasma were introduced into a sealed bag for analysis. The composition and concentration of the gaseous products were analyzed at Wuhan New Radar Gas Co., Ltd, Hubei province, China.

EPR experiments were implemented at room temperature (16–18 °C) using a Bruker EPR A200 X‐band spectrometer (Bruker, Germany). DMPO purchased from Sigma, Shanghai, China, was used to capture the reactive species generated in the CO_2_ reduction system. The DMPO solution was degassed at least twice by performing freeze–pump–thaw cycles using CO_2_ gas. Once the triboelectric plasma process ended, a certain amount of solution was collected through the capillary tube and placed into a quartz nuclear magnetic resonance tube together with the DMPO solution. Then, the tube was put into the EPR equipment for detection and analysis.

### Triboelectric Plasma Simulation

The spatial‐temporal distributions of electron density and electron energy density of the triboelectric plasma were calculated using the 2D PASSKEy (PArallel Streamer Solver with KinEtics) code under the cylindrical axis. The code solved a set of drift‐diffusion‐reaction equations coupled with Poisson's equation, and detailed mathematical formulas and proofs could be found in the literature.^[^ ^8^, ^22^ ^]^ The code used in this work has been well validated by high resolution experimental measurements of electric field, optical emission, discharge morphology, as well as voltage‐current profiles. Details could be found in the literature for point‐to‐plane discharges, in the literature for surface discharges, and in the literature for point‐to‐point discharges.^[^ ^23^ ^]^ The non‐Maxwellian EEDF, electron swarm parameters, and electron impact reaction rates were obtained with the help of the BOLSIG+ package under the two terms approximation of the Boltzmann equation of electrons.

### Statistical Analysis

All the experiments in this paper were operated at least twice, independently. There were few differences between the two experimental data. The *V*, *I*, *P*_ave_, *r_CO_*, O_2_ generation rate, CO selectivity, *η*_ele − chem_, *η*_mech − chem_, and CO_2_ conversion rate were obtained by averaging the data. All the data were performed using Origin Software (OriginLab Corporation, USA), obtaining the average value and error bar (the standard deviation). EPR spectrum was simulated and analyzed using the biomolecular EPR spectrum software. Triboelectric plasma simulation and analysis were performed using the method previously reported in the literature.^[^ ^22^, ^23^ ^]^

## Conflict of Interest

The authors declare no conflict of interest.

## Supporting information


Supporting Information

## Data Availability

The data that support the findings of this study are available in the supplementary material of this article.
