# Supplementary material for: Triboelectric Plasma CO2 Reduction Reaching a Mechanical Energy Conversion Efficiency of 2.3%
Source: Adv Sci (Weinh). 2022 Jun 9;9(23):2201633. doi: 10.1002/advs.202201633 (PMC9376830; doi:10.1002/advs.202201633)
Supplement: Supplementary file 1 — Supporting Information [file ADVS-9-2201633-s001.pdf]

Supplemental Information

Triboelectric Plasma CO_2 Reduction Reaching a Mechanical Energy Conversion Efficiency of 2.3%

Sumin Li[1,3], Bao Zhang[1,3], Guangqin Gu[1], Xiaochen Xiang[1], Wenhe Zhang[1], Yifei Zhu[2], Dongyang Fang[1], Jiao Wang[1], Junmeng Cuo[1], Peng Cui[1], Gang Cheng[1,*], Zuliang Du[1,*]

[1] Key Lab for Special Functional Materials, Ministry of Education, National & Local Joint Engineering Research Center for High-efficiency Display and Lighting Technology, School of Materials Science and Engineering, and Collaborative Innovation Center of Nano Functional Materials and Applications, Henan University, Kaifeng 475004, China

[2] Institute of Aero-engine, School of Mechanical Engineering, Xi'an Jiaotong University, Xi'an 710049, People's Republic of China

[3] These authors contributed equally to this work: Sumin Li, Bao Zhang.

[*]To whom correspondence should be addressed. Email: chenggang@henu.edu.cn; zld@henu.edu.cn

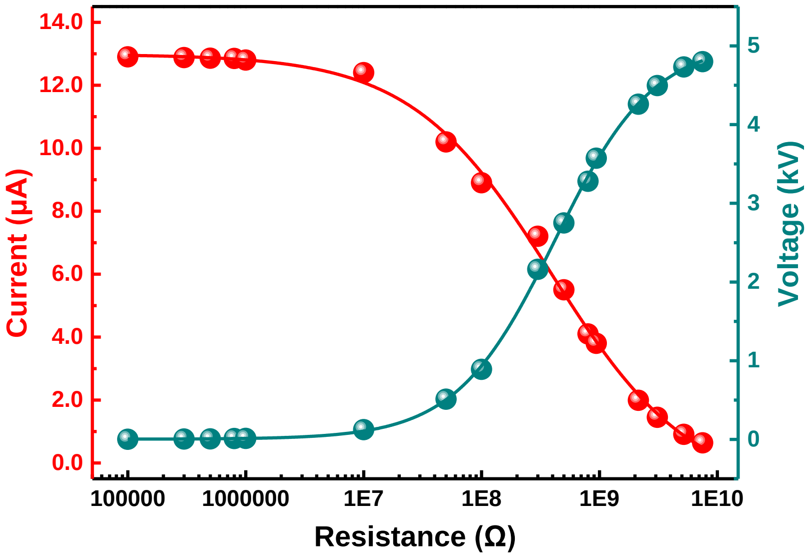

Figure S1 Output characteristics of the triboelectric nanogenerator (TENG) device: open-circuit voltage and short-circuit current generated by the TENG at a rotational speed of 180 rpm.

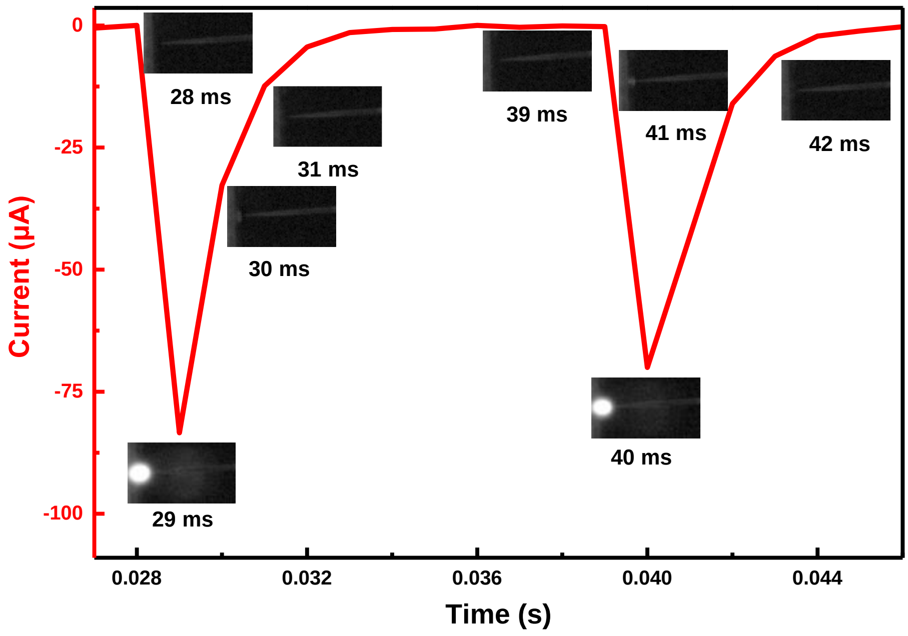

Figure S2 High-speed photographs of triboelectric plasma at different discharge times.

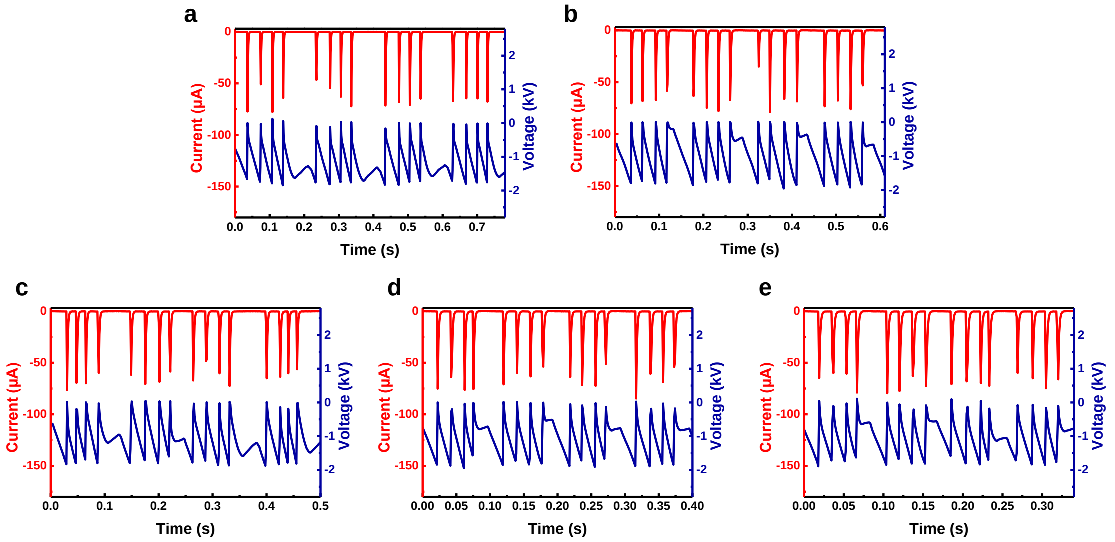

Figure S3 Current and voltage curves of triboelectric plasma at TENG rotational speeds of 60, 90, 120, 180, and 210 rpm. Reaction conditions: discharge distance, 0.8 mm; CO_2 flow rate, 10 mL min^{-1}; room temperature; and atmospheric pressure.

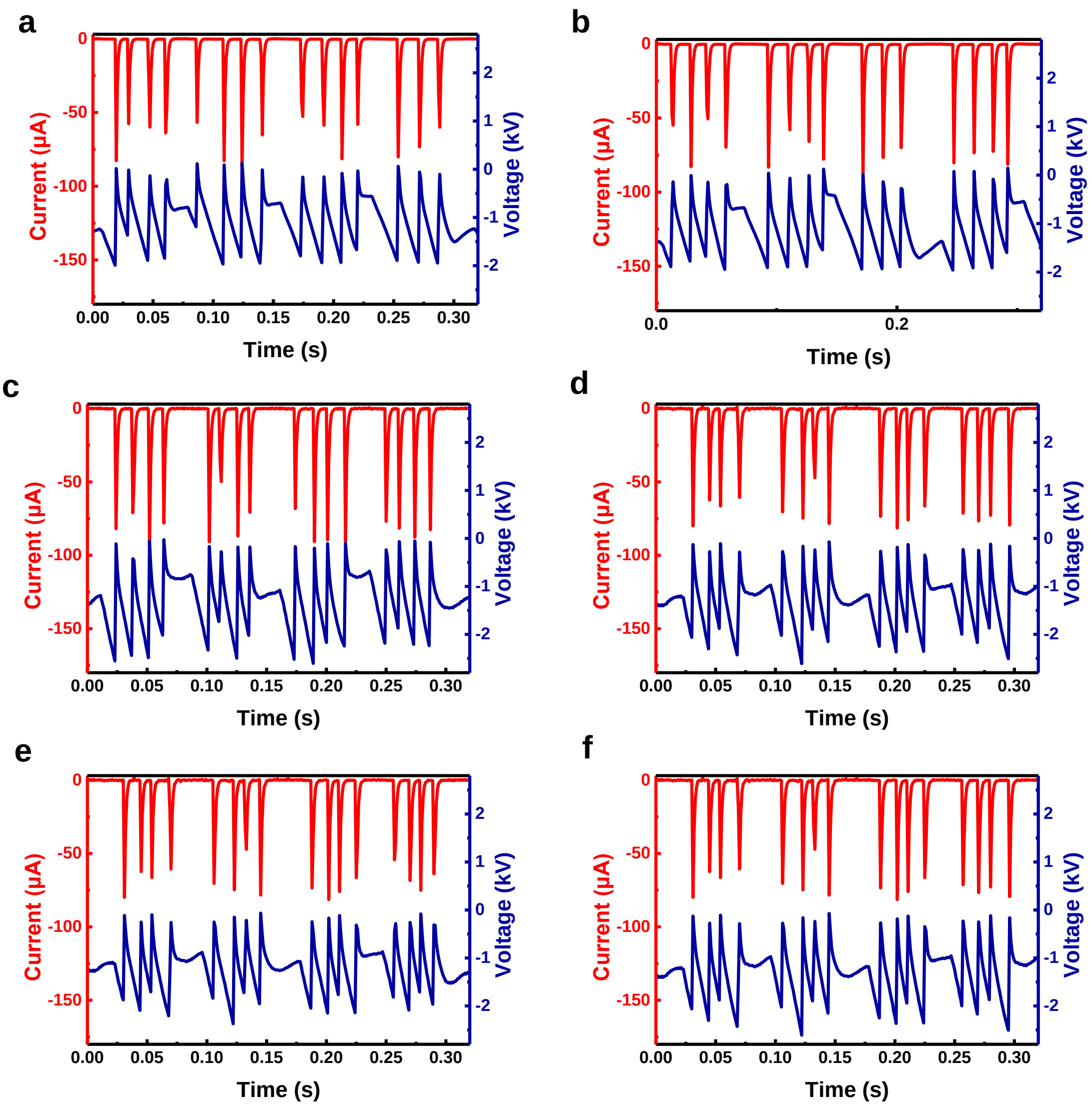

Figure S4 Current and voltage curves of triboelectric plasma at CO_2 flow rates of 0.2, 1.0, 5.0, 7.5, 10, and 12.5 mL min^{-1}. Reaction conditions: TENG rotational speed, 180 rpm; discharge distance, 0.8 mm; room temperature; and atmospheric pressure.

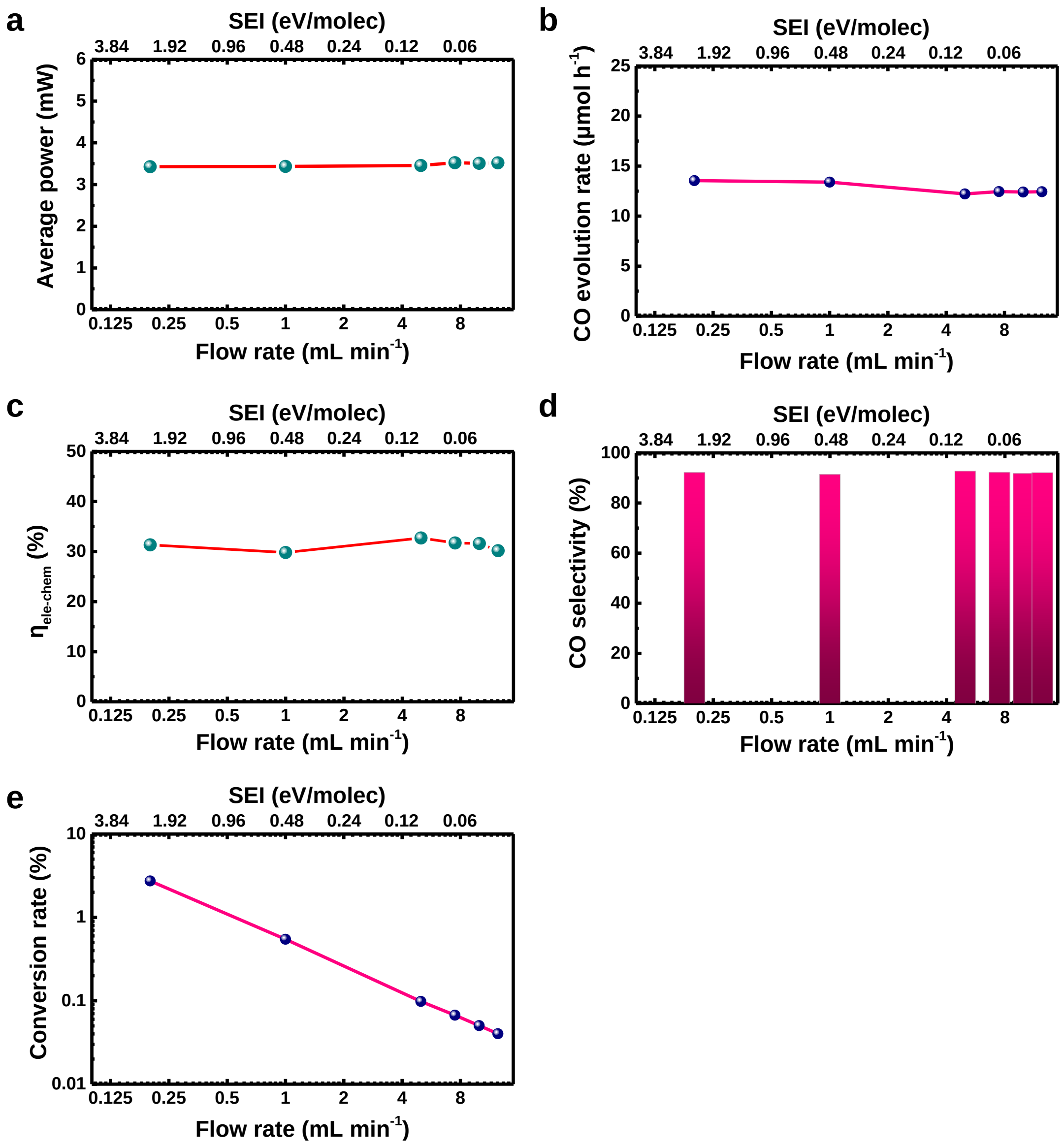

Figure S5 Influence of CO_2 flow rate on the CO_2 decomposition performance. a) Average power versus CO_2 flow rate. b) CO evolution rate versus CO_2 flow rate. c) Conversion efficiency from electrical to chemical energy ($\eta_{ele-chem}$) versus CO_2 flow rate. d) CO selectivity at different CO_2 flow rates. e) CO_2 conversion rate at different CO_2 flow rates. Reaction conditions: TENG rotational speed, 180 rpm; discharge distance, 0.8 mm; room temperature; and atmospheric pressure. Note: SEI is the molar ratio of specific energy input to reaction gas.

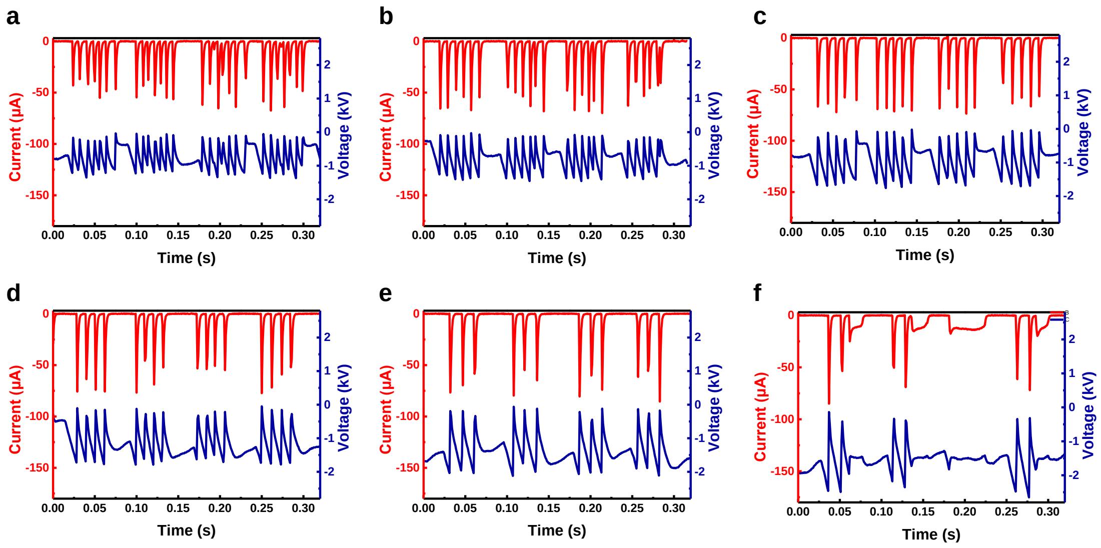

Figure S6 Current and voltage curves of triboelectric plasma at discharge distances of 0.2, 0.4, 0.6, 0.8, 1.0, and 1.2 mm. Reaction conditions: TENG rotational speed, 180 rpm; CO_2 flow rate, 10 mL min^{-1}; room temperature; and atmospheric pressure.

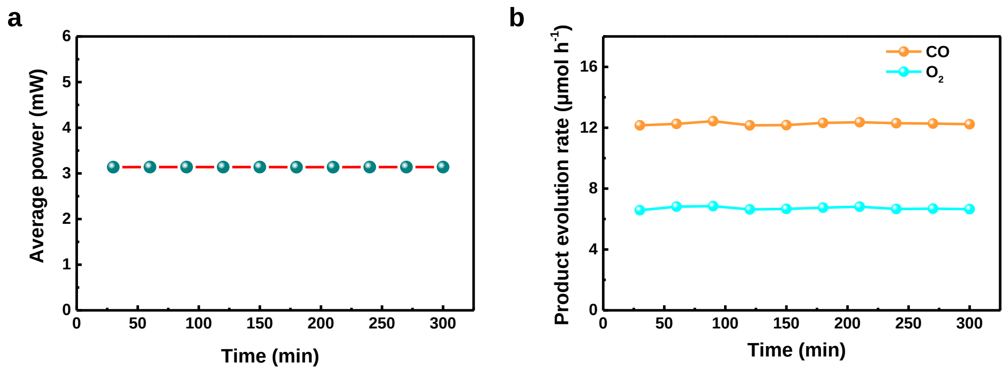

Figure S7 Stability of the CO_2 decomposition performance. a) Average power within 300 min of reaction. b) CO and O_2 evolution rates within 300 min of reaction. Reaction conditions: TENG rotational speed, 180 rpm; CO_2 flow rate, 10 mL min^{-1}; discharge distance, 0.8 mm; room temperature; and atmospheric pressure.

Table S1 Conversion efficiencies from electrical to chemical energy ($\eta_{ele-chem}$) in CO_2 decomposition via different types of nonthermal plasma

References	Plasma types	SEI (eV/molec)	$\eta_{ele-chem}$ (%)
**5b**	Pulsed corona discharge	0.1–0.5	17.0–25.0
**5c**	Pulse corona discharge	1.9–18.7	3.4–10.5
**5d**	Corona discharge	5.6–14.0	3.4–3.9
**12b**	Corona discharge	5.8–46.7	2.0–8.0
**12c**	DBD	7.0×10^{-3}–17.7×10^{-3}	2.2–3.7
**12d**	DBD	0.6–0.8	7.1–9.6
**12e**	DBD	2.8	3.4
**12f**	DBD	4.4–16.8	2.8–3.3
**12j**	DBD	1.5–3.9	12.5–23
**12h**	Nonthermal plasma	2.3	2.2–6.3
**12j**	Nanosecond-pulsed discharge	0.1–0.3	7.3–11.5
**This work**	Multiple pulse, flow-type triboelectric plasma	1.33×10^{-2}–3.32×10^{-2}	30.5–31.9

DBD: dielectric barrier discharge; SEI: the molar ratio of specific energy input to reaction gas.

Table S2 CO selectivities in CO_2 decomposition via different types of nonthermal plasma

References	Plasma types	$\eta_{ele-chem}$ (%)	CO selectivities (%)
**5d**	Pulse corona discharge	2.1–3.1	16.0–20.0
**8a**	Corona-type triboelectric plasma	5.2	100
**12a**	Pulsed corona discharge	4.4	42.0
**12b**	Corona discharge	2.0	90.0
**12h**	Nonthermal plasma	2.2–6.3	58.0–60.0
**12j**	Nonthermal plasma	14.3–19.4	36.4–39.0
**12k**	Pulse corona discharge	6.8	80.0
**12l**	Pulse corona discharge	0.9	60.0
**This work**	Multiple pulse, flow-type triboelectric plasma	30.5–31.9	90.8–92.4

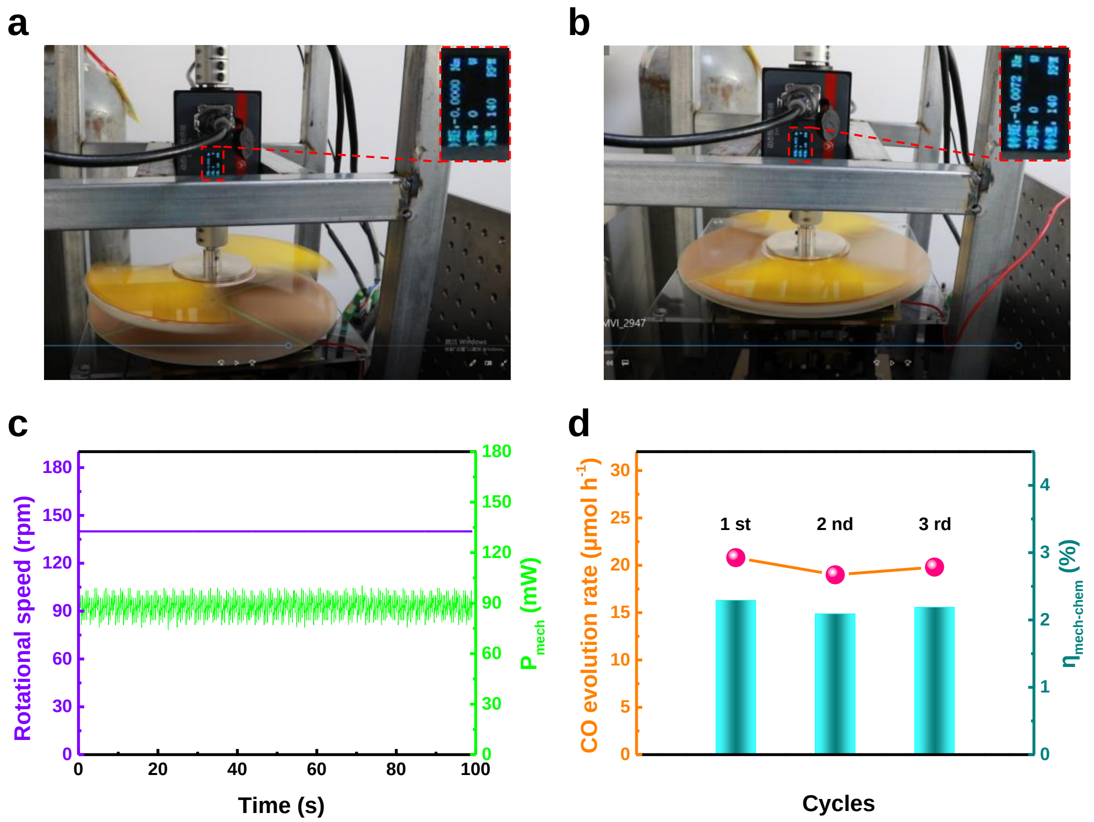

Figure S8 Conversion efficiency from mechanical to chemical energy ($\eta_{mech-chem}$) in the triboelectric plasma-driven CO_2 reduction system. a) Separated triboelectric layers of the TENG. b) Contacting triboelectric layers of the TENG. c) Measured TENG rotational speed and power of mechanical energy (P_{mech}) provided for the TENG with contacting triboelectric layers. d) CO_2 reduction activities and energy efficiencies ($\eta_{mech-chem}$) at 3 runs.

Table S3 CO_2 decomposition driven by mechanical energy

Reference	Sources of mechanical energy	Methods of CO_2 reduction	Products	Products evolution rate (μmol h^{-1})	$\eta_{mech-chem}$ (%)
**7**	Ocean wave energy	Electrocatalysis	HCOOH	0.12	0.05 (measured value)
**8a**	Mechanical energy	Corona-type triboelectric plasma	CO	0.6	1.2 (theoretical value)
**This work**	Mechanical energy	Multiple pulse, flow-type triboelectric plasma	CO	16.8	2.30 (measured value)

Table S4 Dissociation pathways for triboelectric plasma-driven CO_2 decomposition

Entry	Dissociation pathway	Reaction equation	Energy barriers (eV)
$CO_{2(D)}$	Electronic excitation dissociation	$e + CO_2(^1\Sigma^+) \rightarrow CO(a^3\Pi) + O(^3P)$	10.5
$CO_{2(I)}$	Election impact ionization	$e + CO_2 \rightarrow CO_2^+(X^2\Pi_g) + 2e$	13.8
		$CO_2^+ \rightarrow CO^+ + O$	0.4
$CO_{2(V)}$	Vibrational excitation dissociation	$e + CO_2(^1\Sigma^+) \rightarrow CO_2^*(^3B_2) \rightarrow CO(^1\Sigma^+) + O(^3P)$	5.5
$CO_{2(E)}$	Electron attachment dissociation	$e + CO_2 \rightarrow CO_2^-$	0.8
		$CO_2^- \rightarrow CO + O^-$	3.9

Table S5 Main transition peaks detected by optical emission spectroscopy in the CO_2 dissociation via triboelectric plasma

Species	Wavelength (nm)	Transition	Reaction	Entry
**O**	777.5, 844.7	3p→3s	$e + CO_2 \rightarrow CO(a^3\Pi) + O(^3P)$	$CO_{2(D)}$
			$e + CO_2 \rightarrow CO + O(^3P)$	$CO_{2(V)}$
**CO**	206–258	$a^3\Pi \rightarrow X^1\Sigma$	$e + CO_2 \rightarrow CO(a^3\Pi) + O(^3P)$	$CO_{2(D)}$
**CO_2^+**	351.1, 367.4, 434.2	$X^2\Pi \rightarrow A^2\Pi$	$e + CO_2 \rightarrow CO_2^+(X^2\Pi_g) + 2e$	$CO_{2(I)}$

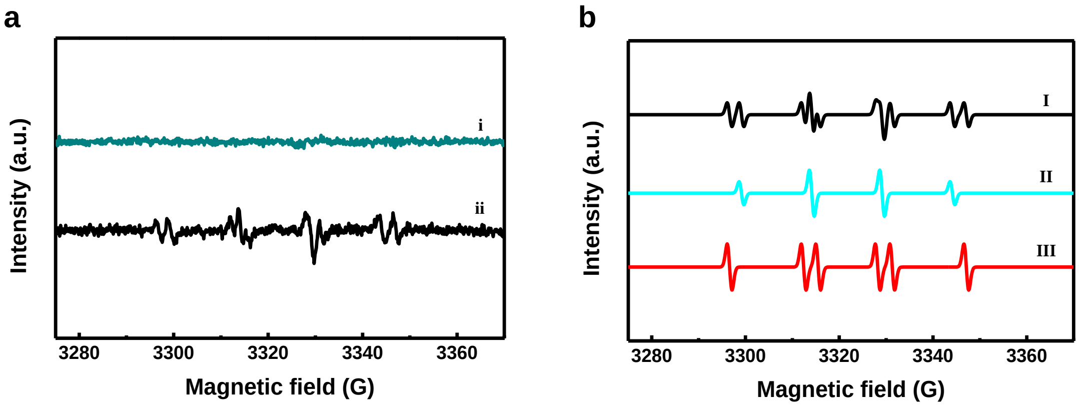

Figure S9 Detection of CO_2-derived reactive species in the triboelectric plasma. a) Curves i and ii are the electron paramagnetic resonance (EPR) spectra without and with the triboelectric plasma, respectively. b) Simulated EPR spectra of the reactive species, where curves I–III are the simulated sum of EPR spectra, the simulated EPR spectra of DMPO–OH, and the simulated EPR spectra of DMPO−CO_2, respectively.

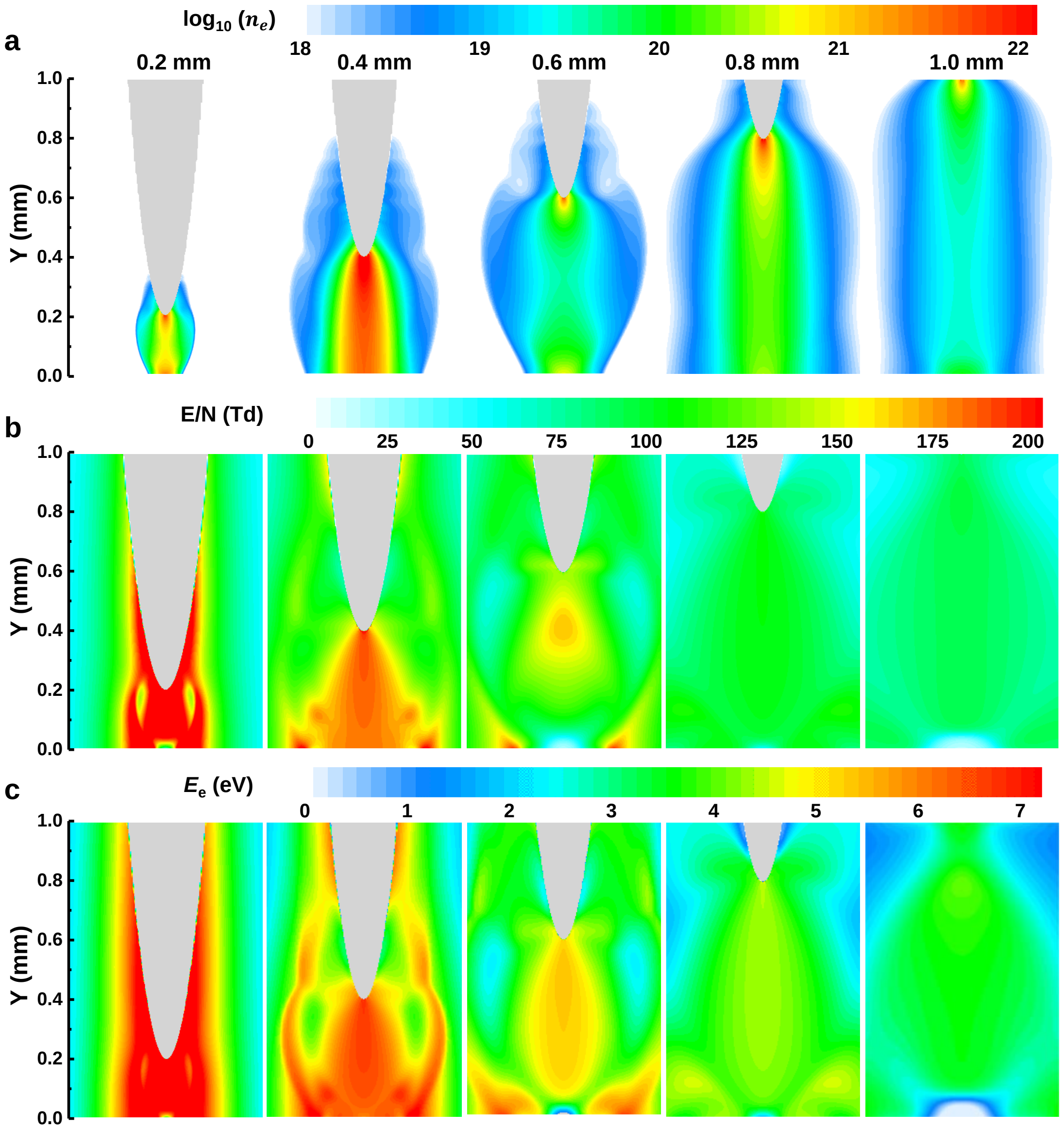

Figure S10 Triboelectric plasma simulation at different discharge distances at 4 ns. a) Evolution of the electron density (n_e) distribution. b) Evolution of the reduced electric field intensity (E/N) distribution. c) Evolution of the average electron energy (E_e) distribution.

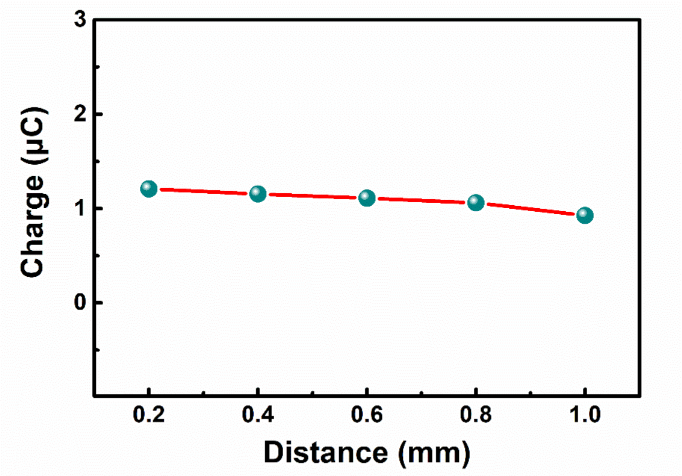

Figure S11 Total amount of charge transferred per cycle of TENG when the gas was discharged at different discharge distances.

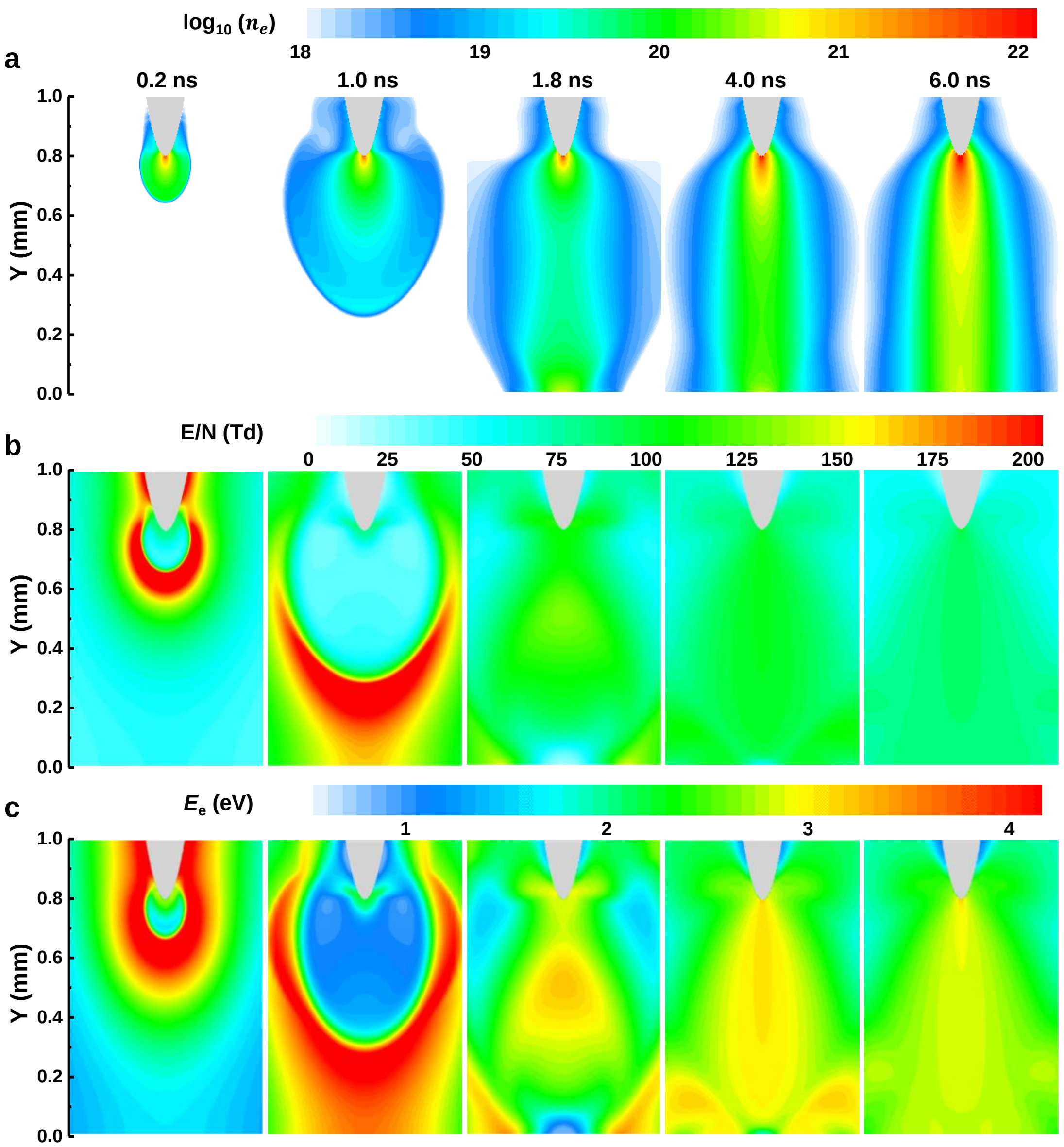

Figure S12 Triboelectric plasma simulation at a discharge distance of 0.8 mm. a) Time evolution of the electron density (n_e) distribution. b) Time evolution of the reduced electric field intensity (E/N) distribution. c) Time evolution of the average electron energy (E_e) distribution.

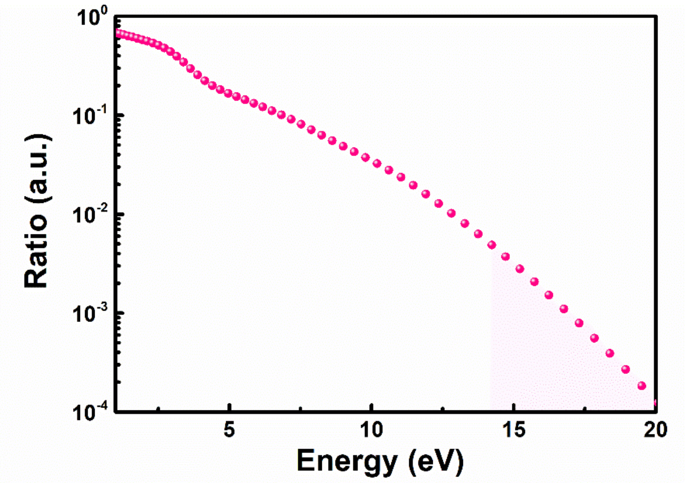

Figure S13 Electron energy distribution function (EEDF) of the point at 8 μm from the tip at an evolution time of 6.0 ns and a discharge distance of 0.8 mm.

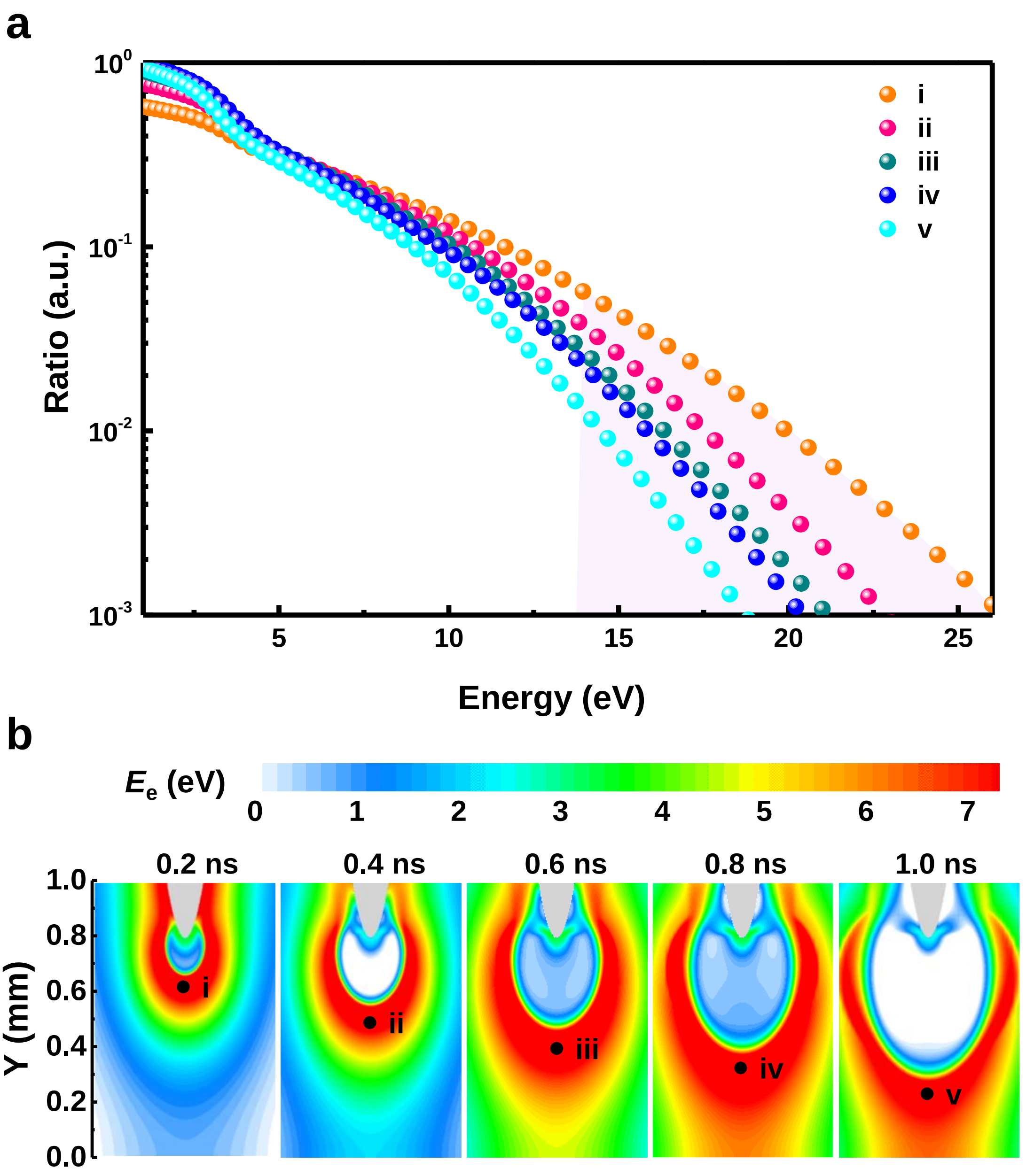

Figure S14 a) Electron energy distribution function (EEDF) at different points (i–v) at a discharge distance of 0.8 mm. b) Time evolution of the average electron energy (E_e) distribution at a discharge distance of 0.8 mm.

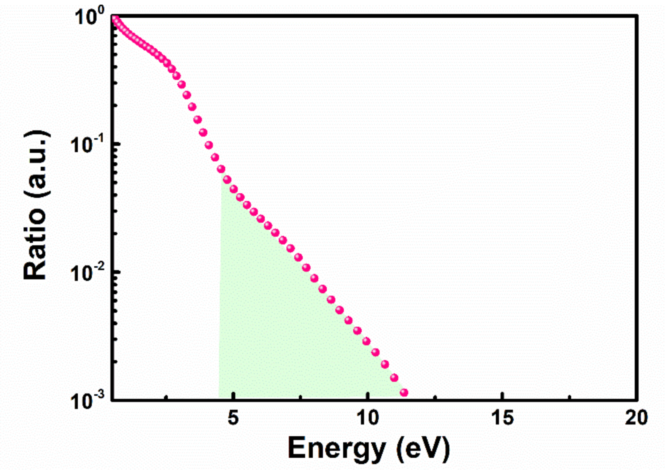

Figure S15 Electron energy distribution function (EEDF) of the point at 0.1 mm from the tip at an evolution time of 6.0 ns and a discharge distance of 0.8 mm.

Table S6 Effective energy levels of CO_2 included in the model

Model notation	States	Energy (eV)	Model notation	States	Energy (eV)
**CO_2**	000	0.00	CO_{2V8}	008	2.24
**CO_2^-**	000	0.80	CO_{2V9}	009	2.51
**CO_2^+**	010	13.8	CO_{2V10}	010	2.77
**CO_{2Va}**	010	0.08	CO_{2V11}	011	3.03
**CO_{2Vb}**	020 + 100	0.17	CO_{2V12}	012	3.29
**CO_{2Vd}**	030 + 110	0.25	CO_{2V13}	013	3.55
**CO_{2Vd}**	040 + 120 + 200	0.23	CO_{2V14}	014	3.80
**CO_{2V1}**	001	0.29	CO_{2V15}	015	4.04
**CO_{2V2}**	002	0.58	CO_{2V16}	016	4.29
**CO_{2V3}**	003	0.86	CO_{2V17}	017	4.53
**CO_{2V4}**	004	1.14	CO_{2V18}	018	4.77
**CO_{2V5}**	005	1.43	CO_{2V19}	019	5.01
**CO_{2V6}**	006	1.70	CO_{2V20}	020	5.24
**CO_{2V7}**	007	1.97	CO_{2V21}	021	5.47

The levels denoted by letters correspond to the symmetric modes of vibration and the levels denoted by numbers correspond to the asymmetric mode of vibration.

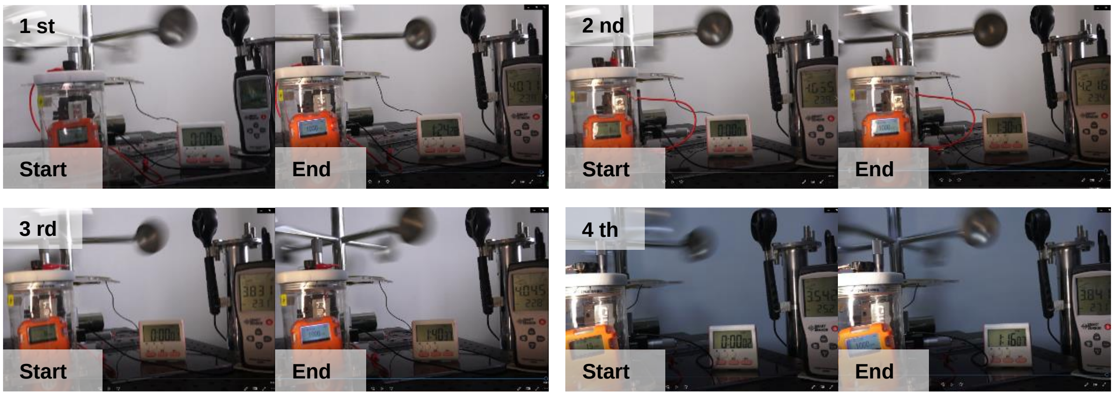

Figure S16 Four field tests of triboelectric plasma driven by mechanical energy in a natural environment.

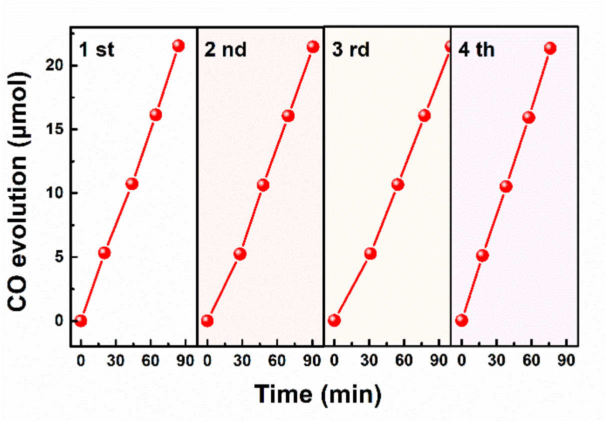

Figure S17 CO yield as a function of time in a field experiment.
